# Health litigation and cancer survival in patients treated in the public health system in a large Brazilian city, 2014–2019

**DOI:** 10.1186/s12889-023-15415-2

**Published:** 2023-03-21

**Authors:** Mônica Silva Monteiro de Castro, Gabriela Drummond Marques da Silva, Iara Veloso Oliveira Figueiredo, Wanessa Debôrtoli de Miranda, Helvécio Miranda Magalhães Júnior, Fausto Pereira dos Santos, Rômulo Paes de Sousa

**Affiliations:** 1 René Rachou Institute, Oswaldo Cruz Foundation (IRR - Fiocruz), Av. Augusto de Lima, 1715 - Barro Preto, Belo Horizonte, MG 30190-002 Brazil; 2 Nursing School, Universidade Federal de Minas Gerais (UFMG), Belo Horizonte, Brazil

**Keywords:** Judicial actions, Public health, Brazil, Cancer drugs, Survival analyses, Cancer

## Abstract

**Background:**

Litigation for health care, also known as health judicialization, is frequent in Brazil. It involves recourse to the court system to access health services. The study aimed to evaluate whether cancer patients in Belo Horizonte, Minas Gerais, Brazil, increased their overall survival by increasing access to certain drugs or treatments through litigation, controlling for the effect of demographic and disease-related variables.

**Methods:**

A retrospective cohort study was conducted. Patients with breast, prostate, brain, lung, or colon cancers from 2014 to 2019 were included. Survival analysis was performed using the Cox proportional hazards model.

**Results:**

In the multivariate analysis, litigation was significantly associated with increased survival in cancers of breast (HR = 0.51, 95%CI 0.33–0.80), prostate (HR = 0.50, 95%CI 0.30–0.85), colon (HR = 0.59, 95%CI 0.38–0.93), and lung (HR = 0.36, 95%CI 0.22–0.60). Five-year survival rates of patients who sued for treatment were 97.8%, 88.7%, 59.3%, and 26.0%, compared to median survival of 95.7%, 78.7%, 41.2%, and 2.4%, respectively, among patient that did not resort to court action. The study suggests that litigation for access to cancer treatment may represent a step forward in obtaining more effective treatment. This study´s main limitations are the lack of patients´ clinical information for use as control variables and the lack of variables to assess patients´ quality of life. The study also found that many cases involved claims that could have been solved by administrative rather than legal action. Some claims thus reflect the lack of adequate administrative procedures.

**Conclusion:**

When based on scientific evidence, access to new therapies, combined with other technologies already available, can favor patient survival. Access to new therapies through litigation may increase health inequalities since low-income patients have limited access to legal recourse against the State to meet their needs. The timely approval of new effective therapies can mitigate the judicialization of cancer treatment.

## Introduction

Health is considered a constitutional right in more than a hundred countries, including Brazil [1]. However, the fact that this right is written into a constitution is insufficient to guarantee its full enforcement. There are widely varying views on the issue, raising conflict between different stakeholders, including patients, families, attorneys, courts, and the public and private health systems.

Brazil´s Federal Constitution of 1988 defines health as the right of everyone and the duty of the State [2]. The Constitution gave rise to the Unified Health System (SUS), which was regulated in 1990 by Laws 8.080 and 8.142 [3, 4]. The founding principles of the SUS are universality, equity, and comprehensiveness, and the system can be used by all people in the Brazilian territory [5]. Brazil´s Federal Constitution did not assign exclusive responsibility to the government for providing health services. According to Article 199, “health care is open to private enterprises” [5].

Two health systems coexist in Brazil. One is the Unified Health System (SUS), which receives public budget financing and offers universal coverage to the population. The SUS serves approximately 75% of the Brazilian population [6]. The other system is called supplementary, with private financing, consisting of private health plans and insurance, serving some 25% of the population [7]. The 25% of the population that have private health plans also use the SUS in some circumstances, due to lack of private provision of services, such as vaccination, epidemiological and health surveillance, pre-hospital care, care in major disasters, and pandemics, among other.

The SUS has made major advances in its nearly 35 years. The system´s development, expansion, and even limitations offer valuable lessons on ways to roll out universal health coverage in a country like Brazil, which presents enormous social inequalities and insufficient government budget allocation when compared to middle- and high-income countries [2, 8].

In this context, a phenomenon just as complex as the right to health emerged in Brazil, namely recourse by various groups to guarantee this right through the courts, known as health judicialization [9]. Most of the articles published on health judicialization analyse countries like Brazil, Costa Rica, Colombia, Chile, and Argentina. That is, Latin America is the region of the world where health judicialization is most common, with a growing trend since the early 2000s [10–12]. In Brazil, the literature points to exponential growth in health-related lawsuits [13], although there is no consensus on the real size of the phenomenon, given the challenges for quantifying such litigation [14].

Legal actions against the public health system are based on the Federal Constitution and on the legislation related to public law. Legal actions against the health insurance companies are based on the Health Insurance Law nº 9.656/98 or the National Health Agency (ANS) resolutions, and/or on the Civil and Consumer Codes [15]. Therefore, litigations against the SUS are processed and judged by the Public Treasury Courts, and Health Insurance’s litigations are processed and judged by Civil Courts.

Judges cannot guarantee anyone's survival. However, when called upon to decide on a health dispute, to rely their decision considering only on medical prescriptions may produce misjudge errors. It is prudent to consider public policies and scientific protocols, and eventually use technical advice from health professionals. Health judicialization is not just moved by patients’ interests, other interests also play a significant role, such as those from the pharmaceutical industries and other actors in the market chain. Studies have identified a possible relationship between the pharmaceutical industry, medical prescribers, and law firms. In some cases, the same attorneys and physicians appear repeatedly in lawsuits filing for new and high-cost drugs [16]. There are also cases in which a single physician signs dozens of legal claims for the same drug [17, 18]. Scientific research and legal aid are also financed by manufacturers of medical supplies and drugs, aimed at their incorporation by the SUS [18]. Furthermore, the speed of advances in pharmaceutical technology – and its long list of failed attempts to meet clinical demands – requires up-to-date information on drug characteristics (efficacy, side effects, cost-effectiveness, advantages over available technologies, etc.), which cannot easily be covered by a legal authority that must decide on the basis of limited technical information provided by the case file.

The influence of the medical-industrial complex is moved by market interests of producers of high-cost innovative medical products. New technologies and scientific discoveries in health are highly lucrative, since they are associated with life itself, a good with incalculable value [19].

The Brazilian public health system faces challenges for its sustainability, especially with the incorporation of new technologies, which involve growing costs in a scenario of limited fiscal room [20]. These challenges have resulted partly from the population´s aging and the growing prevalence of chronic and degenerative diseases, which have particularly increased the expenditures on medical care [21].

Cancer is one of the leading causes of morbidity and mortality in the world, with some ten million new cases and six million deaths per year [22]. Cancer is considered an important global public health problem, with a heavy health, economic, and psychosocial burden [23].

Cancer is the second leading cause of death in Brazil [22]. The National Cancer Institute, INCA [23], predicts that Brazil will experience 625 thousand new cancer cases per year from 2020 to 2022, with a growing budget expenditure [18]. This is occurring in a scenario marked by the appearance of various technological innovations, leading to changes in cancer treatments, with increasingly complex and expensive therapies and drugs [24].

In Brazil, most health-related legal actions involve various therapeutic classes, including antineoplastic medicines (cancer drugs), often involving high costs [10]. Cancer drugs were the therapeutic class most frequently targeted by legal actions in various Brazilian states in recent years [18, 25, 26].

In Brazil, medications for cancer treatment are made available at SUS through hospitals accredited and qualified in oncology and are reimbursed according to predefined medical procedure packages. There are no lists of standardized drugs. Each federative entity has responsibilities in a unique and solidary way with the other entities in accordance with the national oncological policy [27].

One reason leading patients to resort to litigation is the lack of supply of cancer treatments and drugs that have not been incorporated by the public sector due to their high cost. When faced by an administrative refusal to supply such treatments, cancer patients can resort to the courts to enforce their right, through urgent claims involving injunctions and temporary legal remedies to ensure quick and free access to high-cost treatments [28].

New health technologies require heavy research investments, and they increase treatment costs when they are incorporated. The global scenario of accelerated growth in these innovative health technologies, especially in cancer drugs, has led to an increase in inequities in access to cancer treatment, creating an urgent need to restructure health care administration [29, 30].

Both the SUS and the private health care system adopt health technology assessment (HTA) processes, but they take different approaches, further increasing the differences in the supply of products and services to users of the public and private systems [20]. In the SUS, the assessment, incorporation, exclusion, or alteration of health technologies is the responsibility of the National Commission for the Incorporation of Technologies in the SUS (CONITEC), which is prepared to receive and evaluate demands for new oncologic treatments [31].

According to Capucho et al. [32] and Lima; Brito; Andrade, [33], relevant advances were obtained with the creation of CONITEC and the resulting evolution in the process of assessment and incorporation of health technologies in Brazil. However, failure to comply with the legal deadlines for the supply of incorporated technologies, especially in cancer cases, can foment health judicialization.

Health plans in the private health system are regulated by the ANS, which determines the minimum limit for coverage, elaborating and publishing the list of health procedures and events (REPS) [34]. The list includes consultations, tests, therapies, and surgeries that comprise mandatory coverage by regulated health plans. The list´s review process, formerly done in two years, currently adopts a maximum of nine months for analysis of technologies, and cancer treatment technologies are to be assessed in four to six months. Technologies that have already been approved for incorporation by the SUS are to be assessed by the ANS within a maximum of two months [35].

The periodic revision of the ANS list of health procedures and events (REPS) fails to bear a direct relationship to the guidelines followed by CONITEC in technology assessment for the SUS, thus maintaining a dichotomy between the two models, public and private [30]. Recently, Law 14.454 of September 21, 2022, determined that the list should only serve as a basic reference for private health plans, and that other procedures should be covered for which there is scientific evidence proven by CONITEC or by renowned international agencies [36]. The main discussion at present is that the list of agencies provides examples without laying down hard and fast rules. Since the law was only enacted recently, it is still too early to tell how the private health care sector will rearrange to respond to this new legislation.

The scientific literature is divided on the relevance of health judicialization for individuals and populations. One side contends that judicialization meets patients´ interests by expanding access to health products and services, while the other side claims that it jeopardizes equity in access to the SUS by allocating a disproportionate share of already scarce budget resources for highly specific demands. Some questions may help understand the trade-off between patients´ rights and equity in access to health: is litigation good for patients? Does it increase their survival? Does it improve their quality of life?

No published studies have assessed the impact of health judicialization on cancer patients´ survival in Brazil or causality between health judicialization in general and health outcomes, such as improvement of health after a litigation [10]. The current study thus aims to help fill one of these knowledge gaps, namely if there was a survival increase in cancer patients that had a litigation against Minas Gerais State. The city of Belo Horizonte, which had a population of 2,530,701 in 2021, has the fifth highest human development index (0.810 in 2010) among all 5.570 Brazilian municipalities and the sixth best performance in the Unified Health System, measured by the SUS performance index (IDSUS) (6.4 in 2012), among Brazil´s 26 state capitals and the national capital of Brasilia [37, 38]. Considering that private health coverage in Greater Metropolitan Belo Horizonte is 36%, one can assume that 64% of the population depend exclusively on the SUS for their health needs [35].

The aim of this study was to assess whether patients living in the city of Belo Horizonte, who had a diagnosis of breast, prostate, brain, lung, or colon cancer from 2014 and 2019, and who were treated by services operated directly or hired by the Unified Health System (SUS) in the municipality had increased overall survival by resorting to legal action against the Minas Gerais State for some cancer treatment, also considering the effect of other available variables.

## Methodology

### Study design

This was a retrospective cohort study. The cases studied in the sample were persons residing in Belo Horizonte, Minas Gerais, Brazil, who had a diagnosis of breast, prostate, colon, lung, or brain cancer from 2014 to 2019, and who were treated in the Unified Health System (SUS) in the municipality of Belo Horizonte.

The study´s target outcome was time elapsed between cancer diagnosis and death, with deaths recorded up to December 6, 2020. The five types of cancer were chosen on basis of the highest incidence in the population (breast, prostate, lung, and colon) and the highest rate of judicialization (all the above plus brain cancer) [39]. Cancer cases were identified by ICD-10 C50 – Malignant Neoplasm of Breast, C61 – Malignant Neoplasm of Prostate, ICD C18 – Malignant Neoplasm of Colon, C34 – Malignant Neoplasm of Bronchi and Lungs, and C71 – Malignant Neoplasm of Brain.

The target variable was presence versus absence of recourse to litigation against the state of Minas Gerais to obtain some health treatment, with the filing date of the suit with the Minas Gerais State Health Department (SES/MG) from 2014 to 2020, controlling for available variables that could affect patients´ survival. These control variables will be described in the next section. It was not possible to access the lawsuits in which the municipal or Federal government was the exclusive defendant.

### Data sources and study variables

The data on health lawsuits were obtained in February 2021 through the Centre for Support for Judicialization (NAJS) of the Minas Gerais Health State Department (SES/MG) and were extracted from the Information System on Management of Pharmaceutical Care, Judicial Module (SIGAF-JUD) [40] SIGAFJUD is a proprietary system used by SES/MG to manage health lawsuits received by Minas Gerais State, as a defendant.

The data on cancer diagnoses and treatments and deaths from cancer and other causes were obtained in December 2020 from the Belo Horizonte Municipal Health Department (SMSA/BH), which extracted them from the information systems on Authorizations for Hospital Admissions (AIH), Authorizations for High-Complexity Procedures (APAC), and Mortality Information System (SIM), all made available by the Ministry of Health of Brazil and under mandatory use by the Unified Health System (SUS).

**Fig. 1:**
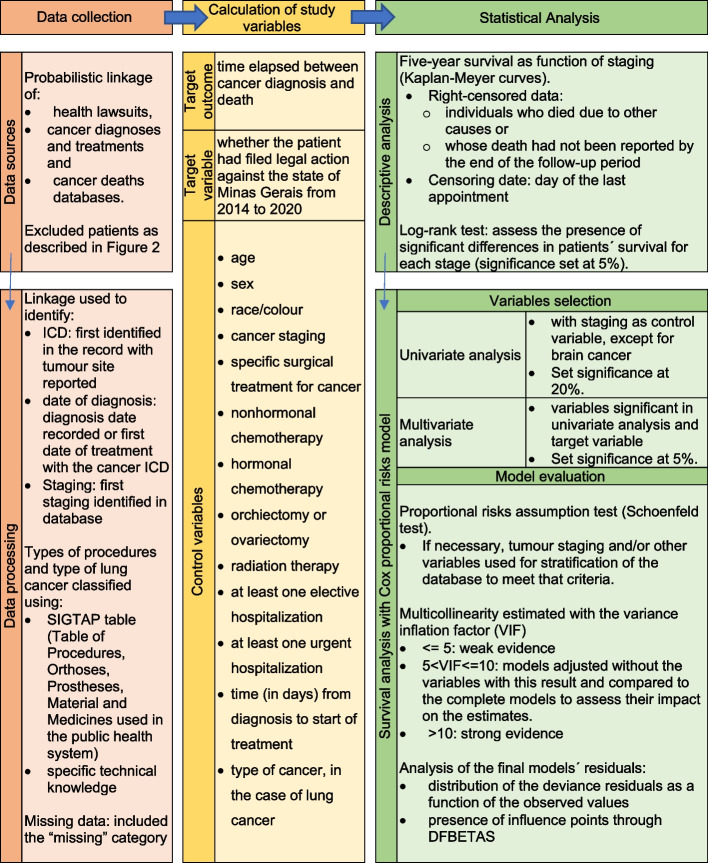
Study Methodology Flowchart

The study´s various databases were linked with probabilistic linkage to identify the ICD-10 (first cancer ICD-10 identified in the patient´s record with the tumour site reported) and date of diagnosis (date of diagnosis recorded in the APAC database, or if not available, first date of treatment with the cancer ICD-10 recorded) (Fig. 1).

The sample excluded patients that were only identified as cancer cases in the Mortality Information System, since they did not have an observation period that could be analysed (*n* = 22,315), patients not residing in Belo Horizonte (*n* = 53,007), since there were no data on use of SUS services by these patients, patients with diagnosis prior to 2014 or after 2019 (*n* = 10,373), and patients with other cancers, not included in the five types chosen for this analysis (*n* = 18,732). The second and fourth groups of patients may be the target of subsequent analyses by this research group.

The control variables were age (continuous, in years), sex (female, male), except for breast cancer in which only cases in women were analysed, and prostate, all of which were in men, race/colour (white vs. non-white), cancer staging (0, 1, 2, 3, 4), record of specific surgical treatment for cancer through identification of the relevant procedures in the Authorizations for Hospital Admissions (AIH) that corresponded to surgeries with curative intent (yes vs. no), record of nonhormonal chemotherapy (no, palliative, and nonpalliative), record of hormonal chemotherapy (no, palliative, and nonpalliative), orchiectomy or ovariectomy, respectively, for prostate and breast cancer (yes vs. no), radiation therapy (no, palliative, and nonpalliative), record of at least one elective hospitalization (yes vs. no), record of at least one urgent hospitalization (yes vs. no), time (in days) from diagnosis to start of treatment (up to 60 days, more than 60 days), where patients who only underwent unspecific cancer treatment were defined as time greater than 60 days, type of cancer, in the case of lung cancer (small cells, non-small cells, not otherwise specified), and finally, whether the patient had filed legal action against the state of Minas Gerais (yes vs. no). Municipality of residence was available in SUS databases as declared by the pacients during cancer treatment. All patients that declared residence in Belo Horizonte at least once were included in the study's sample.

Identification of the types of procedures performed and type of lung cancer was done by the research team using the SIGTAP table (Management System for the Table of Procedures, Orthoses, Prostheses, and Material (OPM) and Medicines in the SUS) and specific technical knowledge. When the data included more than one tumour stage, we used the first staging identified for each patient in the database, based on orientation received from the technical team of the SMSA-BH, responsible for management of patients´ treatment authorizations. Patients with missing data for any control variable were included in the “missing” category.

The study “Health Litigation by Cancer Patients in Greater Metropolitan Belo Horizonte” was approved by the Research Ethics Committee of René Rachou Institute-IRR / Oswaldo Cruz Foundation-FIOCRUZ and by the Research Ethics Committee of Belo Horizonte Municipality Health Department, under case review numbers 3.823.976 and 3.836.359, respectively.

### Statistical analysis

We will present the patients´ distribution according to control variables, the target variable, and outcome by type of cancer.

Five-year survival graphs were built for each type of cancer as a function of staging, using the Kaplan-Meyer estimator. Right-censored data were defined as those for individuals who died from other causes or whose death had not been recorded by the end of the follow-up period. For these patients, the censoring date was the date of the last appointment recorded in the SUS in Belo Horizonte (Fig. 1).

Kaplan-Meyer curves included the survival estimates and 95% confidence intervals for patients whose staging category included at least 20 persons. This cut-off point was chosen empirically, since the estimates for groups with less than 20 persons produced wide confidence intervals (CI), thus hindering the visualization of survival curves. Log-rank test was used to assess the presence of significant differences in patients´ survival for each stage, with significance set at 5%.

Survival analysis was performed with the Cox proportional risks model, which allowed estimating hazard ratios (HR) of patients in each category, controlling for the model´s other variables, as well as this estimator´s 95%CI [41]. Univariate analysis was performed with staging in the models as the control variable, considering this variable´s importance for identification of the cancer´s severity. However, for cancer of the brain, staging is not usually recorded and was thus not used as a control variable. The univariate analysis set significance at 20% and the multivariate analysis at 5%. The target variable was included in the multivariate model, independently of its level of significance in the univariate analysis, to assess its performance with the control variables. The control variables were grouped when the original analytical categories had five observations or less. If this procedure had not been used, some of the models´ estimates would not have converged.

The proportional risks assumption was tested with the Schoenfeld test, which assesses whether the models´ residuals are distributed randomly in time [42]. When necessary to better meet the Cox model´s assumption, especially in relation to judicialization, the tumour staging variable and/or other variables were used for stratification of the database. In models with more than one stratification variable, each variable´s aggregation and thus the number of strata used was the one that met the assumption of proportional risks at least for the judicialization variable and that simultaneously resulted in estimates with smaller standard error.

The models´ multicollinearity was estimated with the variance inflation factor (VIF) [43], defining models with VIF greater than 5 as having moderate evidence of multicollinearity and those with VIF greater than 10 as having strong evidence of multicollinearity. For VIF from 5 to 10, the models were adjusted by excluding the variables with this result and compared to the complete models to assess their impact on the estimates. There was no model with VIF greater than 10. The analysis of the final models´ residuals was performed by verifying the distribution of the deviance residuals as a function of the observed values and the presence of influence points through DFBETAS [44]. DFBETAS quantify each sampling element´s influence on the estimation of the model´s coefficients.

The final multivariate models were represented by forest plots that included the 95%CI of the respective HR [45]. These models were used to estimate the survival function for patients with each type of cancer, according to presence or absence of health-related litigation against the state of Minas Gerais, considering the other variables fixed in the most frequent categories for the respective type of cancer. The analyses were performed with the R software version 4.2.1, using the *survival, survminer,* and *rms* packages [16].

## Results

**Fig. 2:**
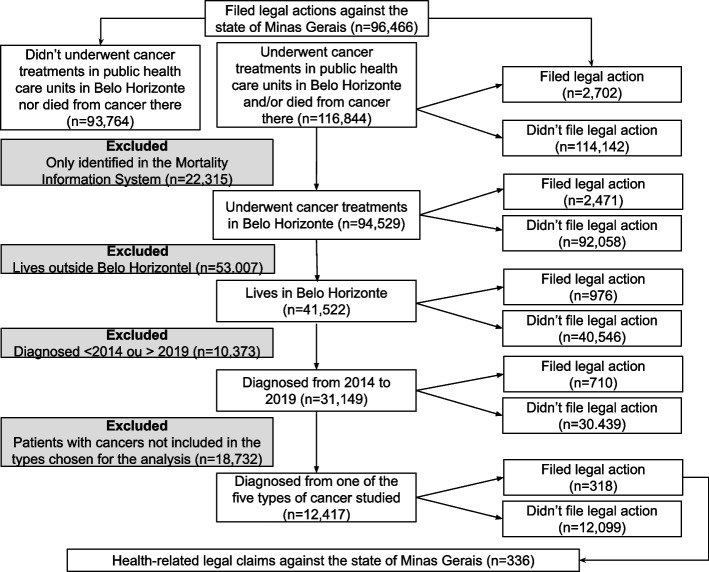
Study´s sampling strategy

We identified 96,466 unique persons recorded in the SIGAF-JUD databased from 2014 to 2019 who had filed legal actions against the state of Minas Gerais, claiming health products and/or services. We also identified 116,844 unique persons who underwent cancer treatments in health care units administered by the Belo Horizonte Municipal Government and/or who died from cancer in that municipality from 2014 to 2020. Non-probabilistic linkage identified 2,702 persons who were in both databases, that is, who filed legal claims against the state and who underwent some type of cancer treatment or died from cancer in the municipality of Belo Horizonte during the target period. After applying the study´s exclusion criteria, we identified 12,417 patients in the sample of whom 318 filed 336 health-related legal claims against the state of Minas Gerais (Fig. 2).

The 12,417 patients included, respectively, 4,614, 3,652, 2,131, 1,354, and 673 cases of cancer of breast, prostate, colon, lung, or brain among persons residing in Belo Horizonte, with data of diagnosis from 2014 to 2019, and treated in the SUS in Belo Horizonte. Of these, 651 (14,1%) persons with breast cancer, 1,954 (53.5%) with prostate cancer, 870 (40.8%) with colon cancer, 561 (41.5%) with lung cancer, and 663 (98.5%) with brain cancer had no information on staging, with the ICD obtained from the AIH or SIM databases. In addition, 51% of breast cancer patients were diagnosed in stages 0 to 2, as were 22% with prostate cancer, 13.3% with colon cancer, and 4.7% with lung cancer (Table 1).

**Table 1:** Profile of the study participants according to type of cancer

Variable	Levels	Type of cancer—n (%)				
		**Breast**	**Prostate**	**Colon**	**Lung**	**Brain**
Age	Mean (Standard deviation)	56.8 (13.4)	67.6 (9.0)	61.5 (14.3)	64.6 (11.3)	48.9 (20.2)
Sex	Female	4614 (100.0)	-	1162 (54.5)	590 (43.6)	312 (46.4)
	Male	-	3652 (100.0)	969 (45.5)	764 (56.4)	361 (53.6)
Race/Color	Non-white	3327 (72.1)	2813 (77.0)	1616 (75.8)	1053 (77.8)	530 (78.8)
	White	1055 (22.9)	558 (15.3)	397 (18.6)	234 (17.3)	116 (17.2)
	Missing	232 (5.0)	281 (7.7)	118 (5.5)	67 (4.9)	27 (4.0)
Cancer staging	0 to 1	1156 (25.1)	150 (4.1)	11 (0.5)	16 (1.2)	1 (0.1)
	2	1186 (25.7)	648 (17.7)	271 (12.7)	47 (3.5)	1 (0.1)
	3	1281 (27.8)	459 (12.6)	488 (22.9)	172 (12.7)	4 (0.6)
	4	340 (7.4)	441 (12.1)	491 (23.0)	558 (41.2)	4 (0.6)
	Missing	651 (14.1)	1954 (53.5)	870 (40.8)	561 (41.4)	663 (98.5)
Surgery for cancer	No	1202 (26.1)	1680 (46.0)	650 (30.5)	1050 (77.5)	214 (31.8)
	Yes	3412 (73.9)	1972 (54.0)	1481 (69.5)	304 (22.5)	459 (68.2)
Nonhormonal chemotherapy	No	3632 (78.7)	3598 (98.5)	876 (41.1)	582 (43.0)	576 (85.6)
	Palliative	206 (4.5)	51 (1.4)	549 (25.8)	623 (46.0)	96 (14.3)
	Nonpalliative	776 (16.8)	3 (0.1)	706 (33.1)	149 (11.0)	1 (0.1)
Hormonal chemotherapy	No	1972 (42.7)	2398 (65.7)	2126 (99.8)	1354 (100.0)	671 (99.7)
	Palliative	323 (7.0)	977 (26.8)	0 (0.0)	0 (0.0)	0 (0.0)
	Nonpalliative	2319 (50.3)	277 (7.6)	5 (0.2)	0 (0.0)	2 (0.3)
Orchiectomy or ovariectomy	No	4594 (99.6)	3212 (88.0)	-	-	-
	Yes	20 (0.4)	440 (12.0)	-	-	-
Radiation therapy	No	1631 (35.3)	2295 (62.8)	2052 (96.3)	912 (67.4)	395 (58.7)
	Palliative	266 (5.8)	386 (10.6)	71 (3.3)	300 (22.2)	41 (6.1)
	Nonpalliative	2717 (58.9)	971 (26.6)	8 (0.4)	142 (10.5)	237 (35.2)
Elective hospitalization	No	3619 (78.4)	2847 (78.0)	1857 (87.1)	1293 (95.5)	586 (87.1)
	Yes	995 (21.6)	805 (22.0)	274 (12.9)	61 (4.5)	87 (12.9)
Urgent hospitalization	No	1295 (28.1)	1391 (38.1)	269 (12.6)	118 (8.7)	93 (13.8)
	Yes	3319 (71.9)	2261 (61.9)	1862 (87.4)	1236 (91.3)	580 (86.2)
Time (in days) from diagnosis to start of treatment	Up to 60 days	2417 (52.4)	1692 (46.3)	1492 (70.0)	736 (54.4)	470 (69.8)
	More than 60 days	2197 (47.6)	1960 (53.7)	639 (30.0)	618 (45.6)	203 (30.2)
Type of lung cancer	Non-small cells	-	-	-	504 (37.2)	-
	Small cells	-	-	-	95 (7.0)	-
	Not specified	-	-	-	755 (55.8)	-
Filed legal action against the state	No	4541 (98.4)	3579 (98.0)	2072 (97.2)	1310 (96.8)	604 (89.7)
	Yes	73 (1.6)	73 (2.0)	59 (2.8)	44 (3.2)	69 (10.3)
Died from the cancer analyzed	No	4004 (86.8)	3289 (90.1)	1615 (75.8)	580 (42.8)	511 (75.9)
	Yes	610 (13.2)	363 (9.9)	516 (24.2)	774 (57.2)	162 (24.1)
Total		4614(100)	3652(100)	2131(100)	1354(100)	673(100)

Mean age of patients varied from 48.9 years (SD = 20.2) for brain cancer to 67.6 (SD = 9.0) for prostate cancer. Among the types of cancer analysed in both sexes, there was a higher proportion of women (54.5%) in colon cancer and a lower proportion (43.6%) in lung cancer. Approximately 77% of persons were classified as non-white in the overall sample, and breast cancer had the lowest proportion of non-whites (72.1%). Surgery was the most common type of treatment in persons with cancer of breast (73.9%), prostate (54.0%), colon (69.5%), and brain (68.2%), while palliative nonhormonal chemotherapy was the most common type for lung cancer patients (46.0%). Most of the patients, regardless of the type of cancer, did not undergo elective hospitalization and underwent at least one urgent hospitalization. The largest share of urgent hospitalizations occurred in lung cancer patients (91.3%) and the lowest among prostate cancer patients (61.9%). The proportion of patients who initiated treatment more than 60 days after diagnosis varied from 30% in brain and colon cancer to 53.7% in prostate cancer (Table 1).

**Fig. 3:**
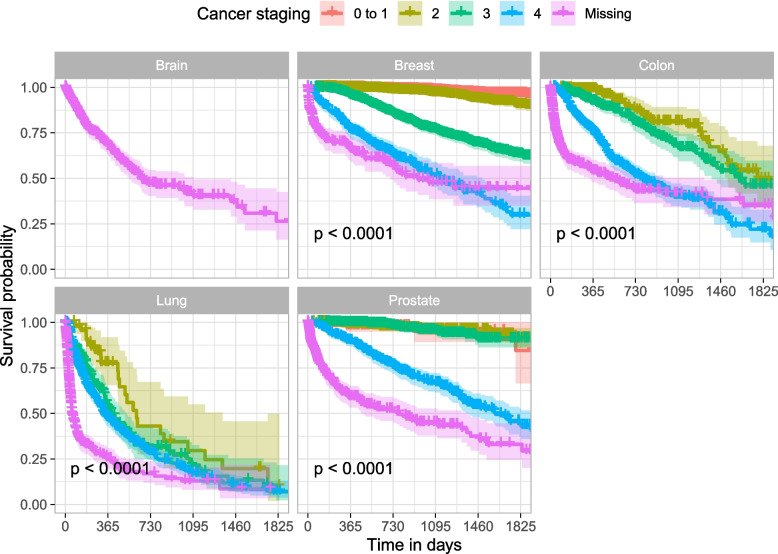
Kaplan-Meyer survival curve for patients with five types of cancer^a,b^. ^a^*p*-value for log-rank test; ^b^Five-year survival: Breast: 76.5%; Prostate: 68.0%; Colon: 34.5%; Lung: 6.4%; Brain: 28.0%

Five-year survival was higher in breast cancer (76.5%) and prostate cancer (68.0%) and lower in colon cancer (34.5%), brain cancer (28.0%), and lung cancer (6.4%). Log-rank test pointed to significant differences in survival according to staging for all types of cancer in which this measure could be calculated (*p* < 0.001). For breast cancer, there was a greater distance between survival for each stage, with estimated five-year survival ranging from 96.3% in stages 0 and 1 to 29.9% in stage 4. In general, patients with no information on staging had low survival, with values equal to or lower than stage 4 (Fig. 3).

In the univariate analysis, considering 20% significance, judicialization was only significant for cancer of lung (HR = 0.40, 95%CI 0.25–0.65) and colon (HR = 0.66, 95%CI 0.42–1.04). As for control variables, the risk of death increased with age for persons with cancer of breast (HR = 1.01, 95%CI 1.00–1.01), prostate (HR = 1.03, 95%CI 1.02–1.04), lung (HR = 1.01, 95%CI 1.00–1.02), and brain (HR = 1.03, 95%CI 1.02–1.04); male gender increased the risk of death among persons with cancer of colon (HR = 1.29, 95%CI 1.09–1.54) and lung (HR = 1.26, 95%CI 1.09–1.46); and white race decreased the risk of death for persons with breast cancer (HR = 0.81, 95%CI 0.66–0.99) (Table 2).

**Table 2:** Harzard ratios for time to death by type of cancer using univariate Cox regression^a^

Variable	Levels	Type of cancer																			
		**Breast**				**Prostate**				**Colon**				**Lung**				**Brain**			
		**HR**	**L95**	**U95**	**p**	**HR**	**L95**	**U95**	**p**	**HR**	**L95**	**U95**	**p**	**HR**	**L95**	**U95**	**p**	**HR**	**L95**	**U95**	**p**
Age	-	1.01	1.00	1.01	**0.07**	1.03	1.02	1.04	**0.00**	1.00	1.00	1.01	0.24	1.01	1.00	1.02	**0.00**	1.03	1.02	1.04	**0.00**
Sex	Female	-	-	-	-	-	-	-	-	1.00	-	-	**0.00**	1.00	-	-	**0.00**	1.00	-	-	
	Male	-	-	-	-	-	-	-	-	1.29	1.09	1.54		1.26	1.09	1.46		0.89	0.65	1.22	0.48
Race/Color	Non-white	1.00			**0.11**	1.00			0.89	1.00			0.24	1.00			0.25	1.00			0.41
	White	0.81	0.66	0.99		0.95	0.70	1.28		0.85	0.68	1.06		1.06	0.88	1.27		1.07	0.73	1.57	
	Missing	0.92	0.61	1.38		0.92	0.59	1.44		1.14	0.77	1.70		0.77	0.54	1.11		1.69	0.82	3.46	
Surgery for cancer	No	1.00			**0.00**	1.00			**0.00**	1.00			**0.00**	1.00			**0.00**	1.00			0.27
	Yes	0.46	0.38	0.54		0.16	0.11	0.23		0.70	0.58	0.83		0.46	0.38	0.57		0.82	0.59	1.16	
Nonhormonal chemotherapy	No	1.00			**0.00**	1.00			**0.00**	1.00			0.37	1.00			**0.00**	1.00			0.00
	Palliative	4.11	3.12	5.40		2.15	1.41	3.27		1.08	0.41	2.87		0.43	0.32	0.57		0.53	0.35	0.78	
	Nonpalliative	5.69	4.58	7.06						0.77	0.28	2.12		0.30	0.20	0.45					
Hormonal chemotherapy	No	1.00			**0.00**	1.00			**0.00**	-	-	-	-	-	-	-	-	-	-	-	-
	Palliative	0.35	0.27	0.44		0.43	0.30	0.60	-	-	-	-	-	-	-	-	-	-	-	-	-
	Nonpalliative	0.11	0.09	0.14		0.30	0.13	0.71		-	-	-	-	-	-	-	-	-	-	-	-
Orchiectomy or ovariectomy	No	1.00			0.70	1.00			**0.00**	-	-	-	-	-	-	-	-	-	-	-	-
	Yes	1.20	0.50	2.89		1.83	1.47	2.28		-	-	-	-	-	-	-	-	-	-	-	-
Radiation therapy	No	1.00			**0.00**	1.00			**0.00**	1.00			0.71	1.00			**0.14**	1.00			**0.00**
	Palliative	1.31	1.03	1.67		1.03	0.77	1.39	-	1.12	0.79	1.58		0.88	0.73	1.06		0.16	0.06	0.45	
	Nonpalliative	0.43	0.35	0.53		0.28	0.18	0.43		0.70	0.17	2.81		0.78	0.59	1.04		0.61	0.44	0.84	
Elective hospitalization	No	1.00			**0.00**	1.00			**0.00**	1.00			**0.00**	1.00			**0.00**	1.00			**0.00**
	Yes	0.38	0.28	0.51		0.45	0.32	0.63		0.44	0.32	0.62		0.31	0.19	0.50		0.31	0.15	0.63	
Urgent hospitalization	No	1.00			**0.00**	1.00			**0.00**	1.00			**0.02**	1.00			**0.00**	1.00			**0.00**
	Yes	4.30	3.02	6.12		3.05	2.08	4.45		1.48	1.06	2.08		1.76	1.28	2.43		2.48	1.22	5.05	
Time (in days) from diagnosis to start of treatment	Up to 60 days	1.00			0.27	1.00			**0.00**	1.00			0.33	1.00			0.21	1.00			0.67
	More than 60 days	0.91	0.78	1.07		2.40	1.78	3.23		0.91	0.76	1.10		1.10	0.95	1.27		1.08	0.76	1.52	
Type of lung cancer	Non-small cells	-	-	-	-	-	-	-		-	-	-	-	1.00			**0.00**	-	-	-	-
	Small cells	-	-	-	-	-	-	-	-	-	-	-	-	1.57	1.21	2.05		-	-	-	-
	Not specified	-	-	-	-	-	-	-	-	-	-	-	-	1.63	1.35	1.97	-	-	-	-	-
Filed legal action against the state	No	1.00			0.94	1.00			0.23	1.00			**0.06**	1.00			**0.00**	1.00			0.22
	Yes	1.02	0.66	1.58		0.74	0.44	1.24		0.66	0.42	1.04		0.40	0.25	0.65		0.75	0.47	1.20	

*Abbreviations*: *HR* Hazard ratio, *L95* Lower 95% CI bound for the Hazard Ratio, *U95* Upper 95% CI bound for the Hazard Ratio, *p p*-value

^a^Univariate analysis with staging as a control variable (except for brain cancer)

All the treatment variables were significant in this phase of the study for cancer of breast, prostate, colon, and lung, except ovariectomy for breast cancer and nonhormonal chemotherapy for colon cancer. No treatment variable was significant for brain cancer. Having at least one elective hospitalization decreased the risk of death for persons with all the types of cancers, and the effect was higher among persons with lung cancer (HR = 0.31, 95%CI 0.19–0.50). Having at least one urgent hospitalization increased the risk of death for persons with all types of cancer, especially breast cancer (HR = 4.30, 95%CI 3.02–6.12). Time from diagnosis to start of treatment greater than 60 days increased the risk of death in prostate cancer (HR = 2.40, 95%CI 1.78–3.23) (Table 2).

**Fig. 4:**
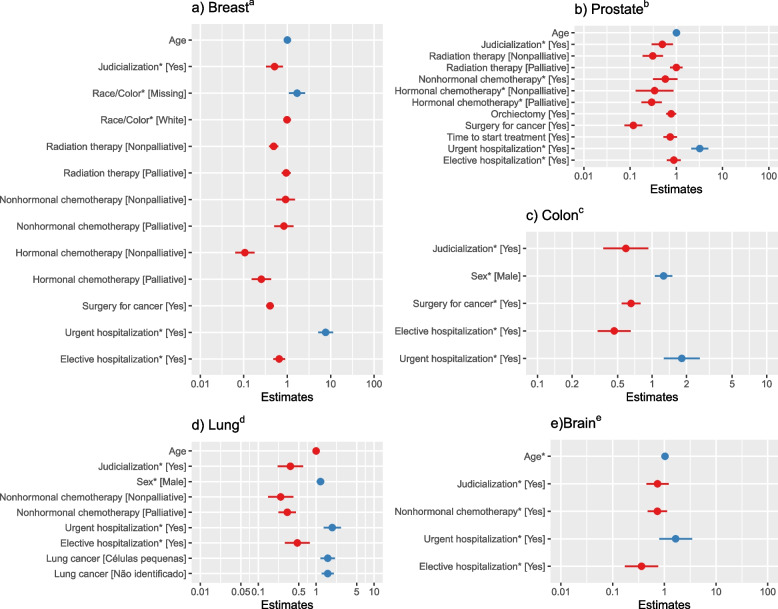
Hazard ratios for time to death by type of cancer using multivariate Cox regression. * met the assumption of proportional risks; ^a^model stratified by staging (0 to 2, 3, 4, missing); ^b^model stratified by staging (0 to 2, 3, 4, missing); ^c^model stratified by staging (0 to 2, 3, 4, missing); ^d^model stratified by staging (0 to 2, 3, 4, missing), radiation therapy (no, palliative, non-palliative), and surgery for cancer (yes, no); ^e^model stratified by radiation therapy (no, palliative, non-palliative)

In the multivariate analysis, the final models for breast, prostate, and colon cancer were stratified by staging; brain cancer was stratified by radiation therapy; and lung cancer by staging, radiation therapy, and surgery. In all these models, the study´s target variable, litigation against the state, met the criteria for Cox proportional risks. Some of the other explanatory variables did not meet the proportional criterion but were maintained for use as control variables (Table 3 and Fig. 4).

**Table 3:** Harzard ratios for time to death by type of cancer using multivariate Cox regression

Variable	Levels	Type of cancer																			
		**Breast**^a^				**Prostate**^b^				**Colon**^c^				**Lung**^d^				**Brain**^e^			
		**HR**	**L95**	**U95**	**p**	**HR**	**L95**	**U95**	**P**	**HR**	**L95**	**U95**	**p**	**HR**	**L95**	**U95**	**p**	**HR**	**L95**	**U95**	***p***
Age	-	1.01	1.00	1.01	**0.01**	1.01	1.00	1.02	0.31					1.00	0.99	1.01	0.74	1.03*	1.02	1.04	**0.00**
Sex	Female									1.00*				1.00*							
	Male									1.26	1.06	1.50	**0.01**	1.18	1.01	1.36	**0.03**				
Race/Color	Non-white	1.00*																			
	White	0.99	0.81	1.21	0.92																
	Missing	1.69	1.10	2.59	**0.02**																
Surgery for cancer	No	1.00				1.00				1.00*											
	Yes	0.40	0.33	0.49	**0.00**	0.12	0.08	0.18	**0.00**	0.66	0.54	0.79	0.00								
Nonhormonal chemotherapy	No	1.00				1.00*								1.00				1.00*			
	Palliative	0.84	0.50	1.40	0.50	0.58	0.32	1.06	0.08					0.33	0.24	0.46	**0.00**	0.73	0.48	1.13	0.16
	Nonpalliative	0.92	0.56	1.51	0.74									0.26	0.16	0.41	**0.00**				
Hormonal chemotherapy	No	1.00				1.00*															
	Palliative	0.25	0.15	0.42	**0.00**	0.29	0.18	0.49	**0.00**												
	Nonpalliative	0.11	0.06	0.18	**0.00**	0.34	0.13	0.87	**0.03**												
Orchiectomy or ovariectomy	No					1.00															
	Yes					0.77	0.61	0.99	**0.04**												
Radiation therapy	No	1.00				1.00															
	Palliative	0.95	0.75	1.21	0.67	1.00	0.73	1.36	1.00												
	Nonpalliative	0.49	0.38	0.62	**0.00**	0.31	0.19	0.51	**0.00**												
Elective hospitalization	No	1.00*				1.00				1.00*				1.00*				1.00*			
	Yes	0.65	0.48	0.89	**0.01**	0.89	0.63	1.25	0.49	0.47	0.34	0.65	**0.00**	0.48	0.29	0.78	**0.00**	0.36	0.17	0.76	**0.01**
Urgent hospitalization	No	1.00*				1.00*				1.00*				1.00*				1.00*			
	Yes	7.67	5.18	11.35	**0.00**	3.21	2.10	4.90	**0.00**	1.82	1.27	2.60	**0.00**	1.94	1.38	2.73	**0.00**	1.65	0.80	3.43	0.18
Time (in days) from diagnosis to start of treatment	Up to 60 days					1.00*															
	More than 60 days					0.74	0.53	1.03	0.07												
Type of lung cancer	Non-small cells													1.00							
	Small cells													1.51	1.15	2.00	**0.00**				
	Not specified													1.54	1.22	1.96	**0.00**				
Filed legal action against the state	No	1.00*				1.00*				1.00*				1.00*				1.00*			
	Yes	0.51	0.33	0.80	**0.00**	0.50	0.29	0.85	**0.01**	0.59	0.38	0.93	**0.02**	0.36	0.22	0.60	**0.00**	0.74	0.45	1.21	**0.23**

*Abbreviations*: *HR* Hazard ratio, *L95* Lower 95% CI bound for the Hazard Ratio, *U95* Upper 95% CI bound for the Hazard Ratio, *p p*-value

^*^Met the assumption of proportional risks

^a^Model stratified by staging (0 to 2, 3, 4, missing)

^b^Model stratified by staging (0 to 2, 3, 4, missing)

^c^Model stratified by staging (0 to 2, 3, 4, missing)

^d^Model stratified by staging (0 to 2, 3, 4, missing), radiation therapy (no, palliative, non-palliative), and surgery for cancer (yes, no)

^e^Model stratified by radiation therapy (no, palliative, non-palliative)

**Fig. 5:**
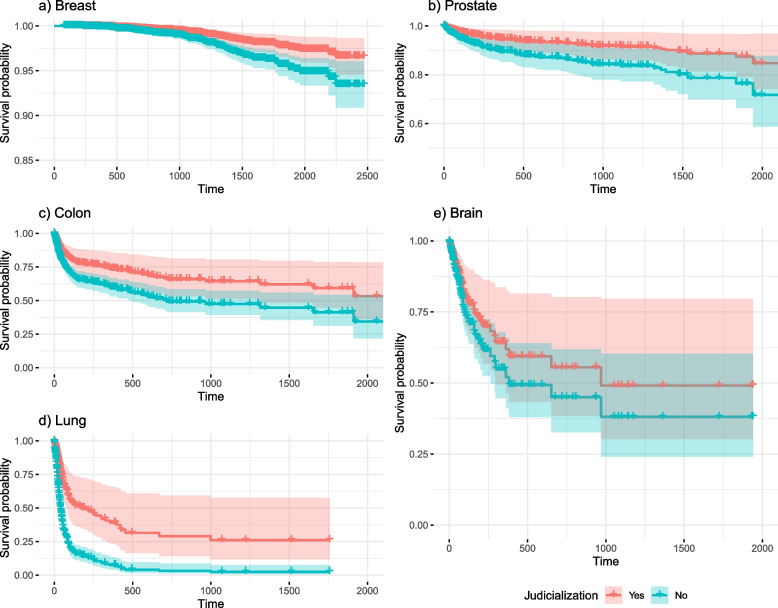
Estimated survival for patients who filed legal claims (versus those who did not)

In the multivariate analysis, the control variables that were significantly associated (5%) with increased survival were: non-palliative radiation therapy (breast: HR = 0.49, 95%CI 0.38–0.62; prostate: HR = 0.31, 95%CI 0.19–0.51), palliative hormonal chemotherapy (breast: HR = 0.25, 95%CI 0.15–0.42; prostate: HR = 0.29, 95%CI 0.18–0.49) or non-palliative hormonal chemotherapy (breast: HR = 0.11, 95%CI 0.06–0.18; prostate: HR = 0.34, 95%CI 0.13–0.87), and surgery (breast: HR = 0.40, 95%CI 0.33–0.49; prostate: HR = 0.12, 95%CI 0.08–0.18; colon: HR = 0.65, 95%CI 0.54–0.79); orchiectomy (HR = 0.77, 95%CI 0.61–0.99) for prostate cancer; palliative (HR = 0.33, 95%CI 0.24–0.46) and non-palliative non-hormonal chemotherapy (HR = 0.26, 95%CI 0.16–0.41) for lung cancer, and at least one elective hospitalization for all types of cancer, except prostate, with the largest effect seen in brain cancer (HR = 0.36, 95%CI 0.17–0.76). The variables associated with decreased survival were a record of at least one urgent hospitalization for all types except brain cancer, with the largest effect in breast cancer (HR = 7.67, 95%CI 5.18–11.34), missing information on race/colour for breast cancer patients (HR = 1.69, 95%CI 1.10–2.60); male gender in colon (HR = 1.26, 95%CI 1.06–1.50) and lung cancer (HR = 1.17, 95%CI 1.01–1.36), and lung cancer not otherwise specified (HR = 1.54, 95%CI 1.22–1.96) or small cell lung cancer (HR = 1.51, 95%CI 1.15–2.00). Risk of death increased with age among brain cancer patients (HR = 1.03, 95%CI 1.02–1.04) (Fig. 5).

Judicialization was significant in the multivariate analysis for breast (HR = 0.51, 95%CI 0.33–0.80), prostate (HR = 0.50, 95%CI 0.30–0.85), colon (HR = 0.59, 95%CI 0.38–0.93), and lung cancer (HR = 0.36, 95%CI 0.22–0.60) (Fig. 5). Five-year survival in patients who filed legal action, considering the other variables fixed in the most frequent categories for each type of cancer, were 97.8%, 88.7%, 59.3%, and 26.0%, respectively, compared to median survival of 95.7%, 78.7%, 41.2%, and 2.4% among patients who did not file legal claims (Fig. 5).

In the assessment of the models´ quality, VIF was less than 5 in all the models analysed except for breast cancer, which showed a VIF of 5.3 for non-palliative hormonal chemotherapy. However, this variable was maintained in the model because it is known to affect breast cancer survival and its exclusion resulted in a model with similar estimated coefficients and significance to those of the complete model for the other variables. Analysis of residuals showed deviance residuals randomly distributed around zero and without evidence of outliers. In addition, DFBETAS values did not present evidence of points of influence on the estimate of the control and target variables´ coefficients.

## Discussion

This study showed that health judicialization was not associated significantly with patient survival in most of the univariate models. However, judicialization showed significant association with four of the five types of cancer studied in the multivariate models. Considering the control variables, judicialization was significant in the multivariate model and decreased the risk of death for persons with four of the five types of cancer (except brain). The largest effect of judicialization was seen in lung cancer, where risk of death was 65% lower, and the smallest significant effect was in colon cancer, were risk of death decreased by 40%.

The different results according to the types of cancer suggest that the effects of health judicialization can vary according to the context studied. Differences related to the interpretation of potential effects from judicialization have also been reported in the literature, where it is viewed alternatingly as a tool for social justice and expansion of access to health services on the one hand, and as a means for the pharmaceutical industry to impose its agendas on health care providers, even in the face of incomplete evidence on the effectiveness of the product being claimed in the lawsuit.

The differences in judicialization´s impact, as observed both in the current study and in the various interpretations reported in the literature, may be related to the patient´s clinical characteristics, such as time to diagnosis, severity, and prognosis; type of product or service claimed in the legal action according to its potential for cure and the evidence of its therapeutic effectiveness; and the flow of the legal process, considering the time between filing the claim until the respective product or service is supplied.

Specifically in relation to legal actions in oncology, we know that many claims are for treatment based on new technologies. However, the incorporation of these technologies in the SUS depends on a process that is often slow, especially for patients at risk of dying, and which begins with the approval by the health regulatory authority (ANVISA) and proceeds to assessment by CONITEC [46].

In oncology, the scope of positive results depends on the integration of a network of specialized care (with medium and high complexity) and non-specialized care (primary care, palliative care, mental health, and others). The various players involved in the system need to understand the influences that affect each patient´s circumstances and lead to changes in clinical practice, allowing the integration of health actions and services, besides the rules and available resources, thereby guaranteeing the rights of persons with cancer [47].

The courts´ tendency to rule in favor of plaintiffs in health lawsuits has led to an increase in legal obligations to provide health services, which in turn requires increased funding for the SUS. However, in the face of budget restraints, compliance with these court rulings tends to exacerbate inequalities on guaranteeing the right to health, since judicialization is a predominantly individual phenomenon, with few class actions [48].

Underfinancing of the SUS was aggravated by Constitutional Amendment 95 (EC/95), enacted in 2016 by the Brazilian Congress. The amendment determines a constrain on primary Federal expenditures until 2036, based on an already declining minimum budget as a proportion of revenue, in which the SUS receives a progressively dwindling share of net current revenue [2]. The amendment has been considered one of the harshest austerity measures implemented in the world due to its duration (20 years), rigidity (altering the Federal Constitution), and scope (failure to permit exceptions). It prevents the allocation of essential resources for promoting public policies needed to implement the fundamental rights set out in the 1988 Constitution [5]. Thus, since 2016 there has been a major cutback in Federal funding for the SUS, resulting in severe financial constraints and major difficulties in public health administration [49].

Court rulings on cancer treatment involve large volumes of financial resources. The impact on budget expenditures to meet health-related claims, mainly for high-cost medicines not previously planned by government agencies interferes in public health care policies already bound by budget earmarking [26, 48].

Santos [48] states that underfinancing of the SUS poses terrible difficulties for guaranteeing the right to health, and thus the real reasons for health services´ insufficiency need to be addressed by the Legislative and Executive, or health judicialization will continue unabated, with no prospects for resolution [48].

The idea of diminishing judicialization neither collide with CF's right to health nor with the citizen's right to judicialize. Contrariwise, public policies of reducing judicialization are looking for guaranteeing the citizen's right without the use of lawsuit for achieving it. Even when disputes are taken to the courts, the judicialization reduction initiatives, such as advising and dispute resolution chambers, can anticipate the legal resolution, reducing time and costs in the legal processes [50].

Our study found that 30% of brain cancer patients and 53.7% of prostate cancer patients started treatment more than 60 days after diagnosis. Two other studies reported similar results. Shafaee et al. [51], in a study of breast cancer in patients in Southeast Brazil and in Texas, found that mean time from diagnosis to the first treatment was more than 60 days. Ribeiro et al. [52], studying uterine cervical cancer in São Paulo, found median time of 190 days from the screening test with an abnormal result to the confirmatory diagnostic test, with only 7% of patients having received the confirmation within 30 days. They also found a median of 81 days from confirmation of diagnosis to start of treatment, with only 44% of patients initiating treatment within 60 days.

Shafaee et al. [51] did not identify a relationship between delayed initiation of treatment and disease relapse or death. However, this situation has the potential to lead to litigation, since a right is being violated, suggesting the need to fund public policies to improve access to cancer screening and treatment [52].

This study´s main limitation is the lack of patients´ clinical and socioeconomical information for use as control variables. We attempted to minimize this bias whenever possible by including in the model the information on staging, race/colour as a socioeconomic indicator, and types of treatment performed (palliative or not) for indirect classification of patients according to the severity of their clinical evolution.

Another important limitation was the fact that we analysed administrative data without the possibility of verification in patients´ medical files and health records. The secondary data source may have led to underestimation of five-year survival in prostate cancer patients (68%) when compared to other studies such as Concord [53], with 91.6%.

A large share of these patients had the date of diagnosis estimated as the date of first surgical procedure as recorded in the Authorization for Hospitalization (AIH), which probably occurred after laboratory diagnosis. However, there is no evidence of differential bias between the categories of target and control variables for measuring survival. To minimize this bias, the study variables were also built on available information in multiple databases. For the other types of cancer, for example, survival mirrored the estimates in the Concord study for the country from 2010 to 2014 (75.2% for breast cancer, 28% brain, and 8.5% lung) [53].

As further limitations, the study lacked variables to allow assessing patients´ quality of life. Thus, it was not possible to know whether increased survival was associated with improved health status or prolonged survival in inadequate health conditions. Often, the use of medication in cancer patients is followed by worse quality of life due to adverse reactions from the medicines [54].

Studies on health judicialization, in general, are based on theoretical and qualitative approach. When present, quantitative works do not go beyond descriptive methods. The production of evidence about judicialization using multiple sources, at a stage that is not usually explored in the literature, especially on the effect of judicialization on the patient's health after the completion of the judicial process, is a strength of this study. In addition, the use of an inferential model to access the effect of legal demands for therapeutics access on cancer survival, controlling for other related factors, is an approach that had not been performed yet.

The association found in this study between judicialization and increased survival in cancer patients cannot be considered a causal relationship, and more studies are needed to test a possible causal association between these phenomena. For example, patients that file legal claims may have different profiles for characteristics not included in the study, such as level of treatment adherence, among others.

Nevertheless, the study´s findings suggest that the public health system should assess the medications and treatments that have been the object of judicialization to review its flows and orient health service providers to revise their clinical protocols. Thus, monitoring of such legal actions by health departments can contribute not only to avoiding new lawsuits but also to potential improvements in the services provided by the SUS to the Brazilian population.

## Conclusion

Living longer is one of the objectives of treatment for various diseases, including cancer. Still, longer survival does not necessarily mean living better. This study adopted survival time since diagnosis as the criterion for beginning to address the topic. Further studies will be needed to analyse the question of quality in this extra time of life.

Legal rulings alone do not create a clinical solution for the patient. The prospects for a favourable outcome to the disease, especially cure, depend on the clinical conditions and the provenly effective treatments that are available at the time.

We hope that this article can contribute to in-depth reflections on the difficulties faced by patients in Brazil´s Unified Health System in accessing adequate care. Based on these reflections, we further hope that the article will favour discussion on the need for public policies to reduce the number of persons who turn to legal action to obtain treatment. The aim is thus to “de-judicialize”, that is, to mitigate litigation of access to health in Brazil.

## Data Availability

The data that support the findings of this study are available from Minas Gerais State Health Department and the Belo Horizonte Municipal Health Department but restrictions apply to the availability of these data, which were used under license for the current study, and so are not publicly available. Data are however available from the corresponding author upon reasonable request and with permission of from Minas Gerais State Health Department and the Belo Horizonte Municipal Health Department.
